# Meltdose Tacrolimus Population Pharmacokinetics and Limited Sampling Strategy Evaluation in Elderly Kidney Transplant Recipients

**DOI:** 10.3390/pharmaceutics16010017

**Published:** 2023-12-21

**Authors:** Jasper Kamp, Tom C. Zwart, Soufian Meziyerh, Paul J. M. van der Boog, Esther E. Nijgh, Koen van Duin, Aiko P. J. de Vries, Dirk Jan A. R. Moes

**Affiliations:** 1 Department of Clinical Pharmacy & Toxicology, Leiden University Medical Center, 2333 ZA Leiden, The Netherlands; j.kamp@lumc.nl (J.K.); t.c.zwart@lumc.nl (T.C.Z.); 2 Transplant Center, Leiden University Medical Center, 2333 ZA Leiden, The Netherlands; s.meziyerh@lumc.nl (S.M.); a.p.j.de_vries@lumc.nl (A.P.J.d.V.); 3 Division of Nephrology, Department of Internal Medicine, Leiden University Medical Center, 2333 ZA Leiden, The Netherlands

**Keywords:** population pharmacokinetics, tacrolimus, prolonged release, kidney transplantation, elderly

## Abstract

Background: Meltdose tacrolimus (Envarsus^®^) has been marketed as a formulation achieving a more consistent tacrolimus exposure. Due to the narrow therapeutic window of tacrolimus, dose individualization is essential. Relaxation of the upper age limits for kidney transplantations has resulted in larger numbers of elderly patients receiving tacrolimus. However, due to the physiological changes caused by aging, the tacrolimus pharmacokinetics (PK) might be altered. The primary aim was to develop a population PK model in elderly kidney transplant recipients. Secondary aims were the development and evaluation of a limited sampling strategy (LSS) for AUC estimation. Methods: A total of 34 kidney transplant recipients aged ≥65 years, starting on meltdose tacrolimus directly after transplantation, were included. An eight-point whole blood AUC_0–24h_ and an abbreviated dried blood spot (DBS) AUC_0–24h_ were obtained. The PK data were analyzed using nonlinear mixed effect modeling methods. Results: The PK data were best described using a two-compartment model, including three transit compartments and a mixture model for oral absorption. The best three-sample LSS was T = 0, 2, 6 h. The best four-sample LSSs were T = 0, 2, 6, 8 h and T = 0, 1, 6, 8 h. Conclusions: The developed population PK model adequately described the tacrolimus PK data in a population of elderly kidney transplant recipients. In addition, the developed population PK model and LSS showed an adequate estimation of tacrolimus exposure, and may therefore be used to aid in tacrolimus dose individualization.

## 1. Introduction

Relaxation of the upper age limits for solid organ transplantation, coupled with improvements in post-transplant survival, have resulted in greater numbers of elderly patients receiving immunosuppressant drugs such as tacrolimus to prevent organ rejection [1,2]. However, aging is associated with numerous physiological changes that might affect both tacrolimus pharmacokinetics and pharmacodynamics.

Currently, tacrolimus is the backbone of most immunosuppressive regimens in transplant recipients [2]. However, tacrolimus is characterized by a narrow therapeutic window and highly variable pharmacokinetics and toxicity (i.e., nephrotoxicity) when overdosed. Moreover, tacrolimus is characterized by a well-studied concentration–effect relationship [3]. These characteristics make tacrolimus a suitable candidate for model-informed precision dosing (MIPD), which is a method that enables individualized dosing based on population pharmacokinetic models [4].

Tacrolimus dose individualization is based on whole blood trough concentrations (C_0_). Although trough concentrations are often considered to be a reliable predictor of the true exposure, defined as the area under the concentration curve (AUC), C_0_/AUC ratios are highly variable between patients [5,6], and can also change within patients depending on the phase after transplantation [6]. Therefore, tacrolimus dose individualization in our center is primarily based on the tacrolimus AUC, rather than on trough concentrations alone. This policy is based on the improved predictive power of tacrolimus AUC compared to trough concentrations when it comes to predicting biopsy-proven acute rejection [7].

Since full AUC_0–24h_ sampling is a resource=consuming process which imposes an additional burden on the patient, limited sampling strategies (LSSs) can be used to estimate the true AUC_0–24h_, based on a limited number of blood samples.

LSSs have been developed and validated for most tacrolimus formulations [8]. However, because of the specific prolonged release profile of meltdose tacrolimus (Envarsus^®^ [9]), conventional tacrolimus limited sampling strategies (i.e., 0, 1, 3 and 0, 2, 3 h) are unsuitable for accurate AUC estimation [10,11]. Moreover, studies reporting LSSs for meltdose tacrolimus are scarce [10,11], and due to the need for late samples, these LSSs are not always practically feasible [11]. In addition, to our knowledge, no LSS validated in de novo elderly kidney transplant populations has currently been published, thus warranting further investigation.

According to the FDA-approved drug label, steady-state concentrations are typically achieved after 7 days [12]. Conventional early sampled tacrolimus trough concentrations, typically drawn 2–4 days after transplantation, might therefore not be representative of the final steady-state tacrolimus exposure. However, definitive data on the time to steady state are lacking, necessitating further investigation. Potentially, model-informed precision dosing could be used to estimate steady-state tacrolimus exposure, based on early sampled AUC data (i.e., sampled in the first 1–2 days after transplantation).

Despite the advantages of limited sampling schemes in terms of clinical feasibility, whole blood samples still need to be drawn in a hospital setting. To solve this problem, alternative (micro)sampling techniques, such as dried blood spot (DBS) sampling, were developed. DBS sampling is a technique that enables patients to draw their own blood samples at home with a simple finger prick [13]. However, this technique and concurrent organization might not be suitable for all elderly patients, necessitating proper screening and training of suitable patients. Therefore, more data on the feasibility of DBS sampling in elderly populations are necessary.

The primary aim of this study is to develop a population pharmacokinetic model of meltdose tacrolimus, for de novo elderly kidney transplant recipients. The secondary objectives were: (i) the development of a limited sampling strategy that is suitable for MIPD for dose individualization in elderly kidney recipients; (ii) to assess the feasibility of DBS sampling in elderly recipients and (iii) to evaluate the time required to reach steady-state PK after the start of meltdose tacrolimus.

## 2. Methods

The study protocol was approved by the Institutional Review Board of the University Medical Center Groningen (METC Groningen 2018/698) following the Declaration of Helsinki. The primary trial was registered in the ClinicalTrials.gov database with registration number NCT03497196 [14]. The current study protocol is an add-on study to the primary trial at the Leiden University Medical Center (LUMC) site only, and was approved by the Institutional Review Board of the LUMC accordingly.

### 2.1. Study Design

The current study is an open-label prospective evaluation study on the pharmacokinetics of meltdose tacrolimus and add-on study to the larger OPTIMIZE trial. For the OPTIMIZE trial, de novo kidney transplant patients aged 65 years or older at the time of transplantation were eligible. Patients were excluded in case of active infection if there was an a priori high risk of developing severe side effects on the study medication and in cases of high or exceptionally low immunological risk. A complete and detailed overview of the inclusion and exclusion criteria can be found in the published OPTIMIZE study protocol [14]. The primary aim of the OPTIMIZE study was to evaluate the use of a low-dose tacrolimus/everolimus/prednisolone immunosuppressive regimen, compared to a standard tacrolimus/mycophenolate/prednisolone regimen in elderly kidney transplant recipients, both after basiliximab induction. The primary endpoint was a successful transplantation, defined as a functioning graft and an eGFR >30 mL/min per 1.73 m^2^ (deceased donor stratum) or >45 mL/min per 1.73 m^2^ (living donor stratum) 2 years after transplantation [14]. All patients received an initial dose of 7 mg meltdose tacrolimus q.d. (Envarsus^®^, Chiesi Farmaceutici S.p.A., Parma, Italy), starting at the day of transplantation. After this, therapeutic drug monitoring was applied, and the doses were individualized to reach the target exposure. In addition, patients received the following immunosuppressive regimen during the study period: (i) mycophenolate mofetil (CellCept^®^, Roche Pharma AG, Grenzach-Wyhlen, Germany) 500 mg b.i.d. and (ii) prednisolone, with an initial dose of 50 mg b.i.d. starting at the day of transplantation. The prednisolone dose was subsequently tapered to 25 mg b.i.d. at day 4 and to 10 mg q.d. after day 4. Finally, the prednisolone dose was tapered to 5 mg q.d. after three months. Since patients with severe systemic infections were excluded from the study, no CYP inhibitors such as fluconazole were prescribed during the study period.

A dataset of approximately 35 subjects was considered to be sufficient for adequate covariate identification. This assumption was based on a previously published study, which reported a retrospective power of 99% to identify a 50% effect of the CYP3A5*1/*3 mutation on clearance [15].

The selection and collection of relevant demographic data, clinical chemistry data and pharmacogenetic data were based on a previously published TDM consensus guideline [3] and on the data availability. Basic demographic data were collected: Age at the time of AUC sampling, body weight, body length, time after transplantation (days), sex, prednisolone dose at the day of AUC sampling, the use of calcium blockers (nifedipin, barnidipin, lercanidipin, diltiazem, verapamil) and potentially delayed gastric passage at the time of AUC sampling (i.e., patients with recorded diabetes). The following clinical chemistry data were collected: hematocrit, serum albumin, serum bilirubin, ASAT, and serum creatinine. Finally, pharmacogenetic data were collected on the CYP3A5 genotype.

### 2.2. Bioanalytical Assay and Blood Sampling

A full whole blood AUC_0–24h_ (T = 0, 1, 2, 3, 4, 6, 8, 12, 24 h after dose intake) was planned in the second week after tacrolimus initiation. In addition, a reduced DBS AUC (T = 0, 1, 2, 3, 6 h after the last dose) was planned at 6 weeks after transplantation for patients that could perform DBS sampling in the outpatient setting. The selection of patients for DBS sampling was based on the discretion of the treating physician. Moreover, additional AUCs obtained after discharge for routine clinical care were included in the dataset. The tacrolimus concentrations were measured using an LC–MS/MS method, validated for both whole blood samples and DBS samples [16]. See the original paper for a complete and detailed overview of the used reagents and LC–MS/MS method validation.

### 2.3. Population Pharmacokinetic Modelling

#### 2.3.1. Structural Model

A nonlinear mixed effect modeling approach was used to develop the population pharmacokinetic model. Initially, one-, two- and three-compartmental models were evaluated to identify the optimal starting model. In addition, a previously developed model, based on a dataset with liver transplant patients receiving meltdose tacrolimus, was used to fit the current dataset [10]. The best models were expanded with oral absorption lag time, oral absorption transit compartments and a mixture model for the absorption lag time. The inter-individual variability (IIV) and inter-occasion variability (IOV) were tested on each disposition parameter as θ_i_ = θ_pop_ × exp(η_IIV_ + η_IOV_), where θ_i_ is the EBE (empirical Bayesian estimate) for individual i, θ_pop_ is the estimated population parameter value and η_IIV_ and η_IOV_ are the log normally distributed IIV and IOV, specific to parameter θ_pop_.

Model selection was based on the following criteria: (i) a significant change in the −2 Log Likelihood function value (∆OFV), where a ∆OFV of −6.63 or greater was considered significant (*p* < 0.01 assuming a *χ*^2^ distribution); (ii) visual improvements in the goodness-of-fit plots; (iii) visual improvements in the Normalized Predicted Distribution Error (NPDE) plots and (iv) evaluation of the model robustness based on bootstrap analysis of the model parameters, with a sample size of 1000 datasets. In addition, the agreement between the AUC_ref_ and the AUC calculated using the trapezoidal rule was evaluated. AUC_ref_ was calculated as (F_po_ × D × 1000)/CL, where F_po_ was the oral tacrolimus bioavailability fixed at 100%, D the meltdose tacrolimus dose in mg for the AUC_ref_ interval and CL the elimination clearance in L/h. Finally, a prediction-corrected VPC (pcVPC) [17] was performed to evaluate the performance of the model by Martial et al. [10] on our dataset.

#### 2.3.2. Covariate Model

The initial covariate selection used for the covariate analysis was based on physiological plausibility and was also based on known covariates as recently reported in a tacrolimus TDM consensus report by Brunet et al. [3]. Potential covariates were tested on model parameters using both univariate analysis and a stepwise covariate building method (scm) [18]. Allometric scaling of the disposition parameters (i.e., CL/F, Q/F, Vc/F and Vp/F) were a priori included in the model based on biological plausibility. The flow parameters CL/F and Q/F were allometrically scaled as θ_i_ = θ_pop_ × (WT/70)^0.75^, where θ_i_ is the parameter for individual i, θ_pop_ the population parameter and WT body weight. The distribution parameters Vc and Vp were allometrically scaled as θ_i_ = θ_pop_ × (WT/70)^1^.

The effect of age, the CYP3A5 genotype, hematocrit, sex, prednisolone dose, calcium blocker usage and time after transplantation were tested on CL. For the stepwise covariate search, selection criteria of *p* < 0.05 and *p* < 0.01 were used for the forward and backward selection steps, respectively. In addition to the statistical improvements in the model after covariate inclusion, the decrease in the IIV of CL and biological plausibility were used to assess the covariate relevance.

#### 2.3.3. Limited Sampling Strategy

The final model was used to evaluate the performance of several LSSs comprising clinically convenient sampling times. In step 1, the model was used to simulate full concentration–time profiles for 1000 virtual 70 kg patients. Herein, the reference AUC (AUC_24ref_) was defined as AUC_24ref_ = (F × D × 1000)/CL, where *F* is the oral bioavailability (fixed to 100%), *D* the drug dose in mg and CL the apparent elimination clearance in L/h. Subsequently, multiple limited sampling strategies were evaluated by estimating the AUC_LSS_ by using reduced datasets that only included the timepoints of the tested LSSs. To ensure clinical feasibility, only LSSs with samples taken between 0 and 12 h after drug administration were considered. The final LSS selection was based on the following parameters: (i) correlation coefficient of the AUC_24ref_ vs. AUC_LSS_ (R^2^); (ii) relative prediction error (%); (iii) absolute prediction error (µg·h/L) and (iv) probability of >20% bias.

#### 2.3.4. Time to Steady State Analysis

A simulation study was performed to assess the time to steady state in our population. The final population pharmacokinetic model was used to simulate 1000 concentration–time profiles for a 70 kg individual receiving 7 mg meltdose tacrolimus q.d. The time to steady state was evaluated using two approaches: (i) graphical evaluation of the mean simulated concentration–time profiles and (ii) calculation of the fraction of the steady state at different times after dosing with the following equation: F_SS_ = IPRED/C_trough 14_ × 100, where F_SS_ is the fraction of the assumed steady-state concentration, IPRED the individual predicted concentration by the model and C_trough 14_ the steady-state trough concentration, defined as the trough concentration at day 14. Since dose adjustments are advised in our hospital when AUC_0–24h_ values are more than ±20% off target, we assumed 90% of the steady state to be sufficient for MIPD purposes. This 20% percent range is based on a maximum coefficient of variation of 15% reported for most biopharmaceutical assays and an additional random variability due to sampling errors.

#### 2.3.5. Software

Data preparation was performed in Microsoft Excel for Microsoft 365 MSO (Version 2208). RStudio version 4.2.1 was used for the data preparation, the graphical evaluation of the models and simulations. Pharmacokinetic data analyses were performed in NONMEM version 7.4.4 and PsN version 5.0.0.

## 3. Results

### 3.1. Demographics

**Figure 1:**
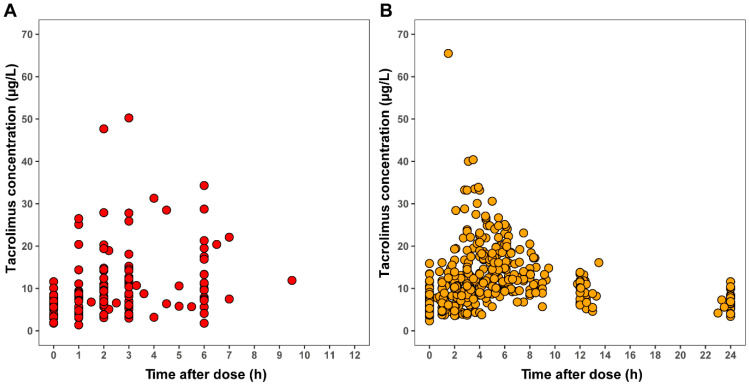
Measured tacrolimus concentrations vs. time after dosing. The red data points show tacrolimus concentrations obtained using DBS sampling (**A**). The orange data points show tacrolimus concentrations obtained using whole blood sampling (**B**).

In total, 36 patients were enrolled in the study. Two patients were excluded from the analyses due to the postponement of their transplantation procedure. Therefore, data from 34 patients could be included in the analyses, with a median age of 71.5 years (IQR: 68.8–73.2) at the time of transplantation. A total of 87 AUCs and 546 samples were obtained (Figure 1). Each patient contributed a median of 2 AUCs (range: 1–5) to the dataset.

The median meltdose tacrolimus dose was 6.0 mg (IQR: 3.25–7.0 mg). Furthermore, 14 patients (41.2%) were reported with diabetes mellitus. Patients received 5–10 mg prednisolone at the time of AUC sampling. However, one patient received a high prednisolone dose of 50 mg per day because AUC sampling was performed earlier than planned (day 5 after tacrolimus initiation). However, since we did not assume a steady state to be reached in all patients, this AUC was retained in the final dataset. Finally, 26 (76.5%) of the patients were non-expressors of CYP3A5, while 8 patients (23.5%) were expressors. A complete overview of the demographics is shown in Table 1.

### 3.2. Dried Blood Spot Sampling Feasibility

The DBS AUCs were obtained in 20 (58.8%) of the patients and amounted to a total of 42.5% of the AUCs in the dataset. An overview of the expected versus observed sampling times is shown in Table S1. Of the 37 DBS sampled AUCs, 20 AUCs (54%) were correctly sampled at T = 0, 1, 2, 3, 6 h after tacrolimus intake, according to the protocol.

### 3.3. Pharmacokinetic Modeling

#### 3.3.1. Structural Model

**Figure 2:**
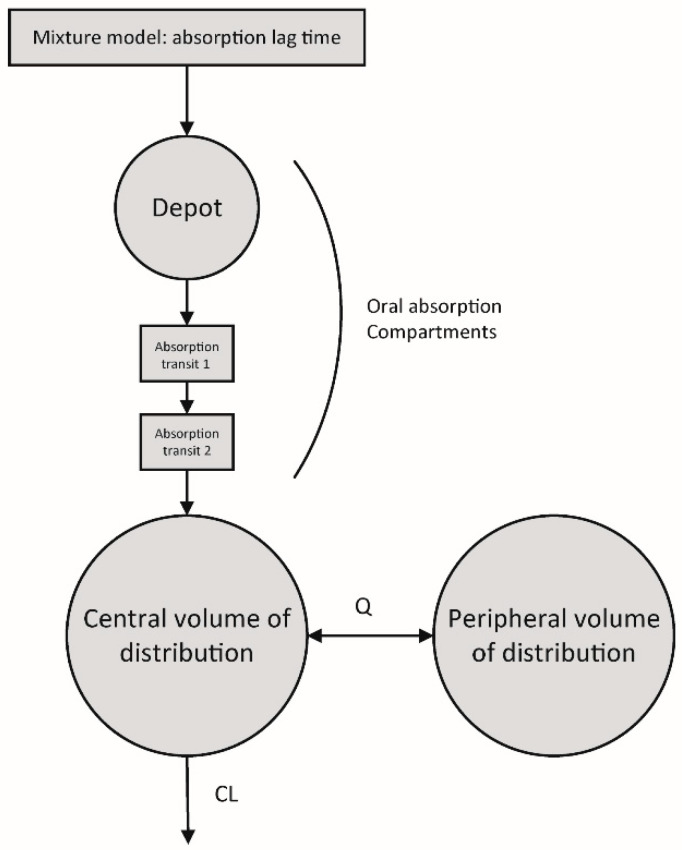
Overview of the structural pharmacokinetic model. First, a mixture model assigns an absorption lag time of either 0 h (no lag time) or an estimation of the lag time, depending on the model fit for that specific individual. After modeling of the absorption lag time, the drug dose is inputted in the depot compartment. Subsequently, the administered dose passes two additional absorption transit compartments, Absorption transit 1 and Absorption transit 2, before reaching the central volume of distribution. The drug is cleared from the central volume of distribution via elimination clearance, CL and intercompartmental clearance, Q.

The data were best described using a structural model including two disposition compartments, with three transit compartments for the oral absorption model. The addition of the three transit compartments resulted in a ∆OFV of −40.5 points, compared to the model without transit compartments. Estimation of the optimal number of transit compartments using the method published by Savic et al. [19] did not further improve the model compared to manual transit compartment testing. The introduction of differential proportional residual errors for a sample matrix (e.g., whole blood vs. DBS) yielded a ∆OFV of −8.2 points. As previously reported, identifiability issues occurred for the peripheral volume of the distribution parameter estimate. We therefore fixed the peripheral volume of the distribution parameter to 500 L/70 kg, based on the literature’s values [10,20]. Introducing IOV on clearance improved the model by 330 points. However, evaluation of eth individual model fits showed slight misfits in the absorption phase of some individuals. This suggested differential absorption kinetics in our population, caused by differences in lag time. We therefore further expanded the model with a mixture model, assigning either no absorption lag time or lag time to everyone, based on the optimal model fit. The addition of the mixture model showed to improve the previously observed misfits in the absorption phase and caused a further reduction in the OFV of −46 points. A graphical representation of the base model structure is shown in Figure 2.

The population parameter estimates (bootstrap 95% CI (Confidence Intervals)) of the final model were: CL/F and Q/F: 19.6 (16.4–22.8) and 74.9 (55.8–86.5) L/h at 70 kg, respectively; Vc/F: 123 (65–273) L per 70 kg; Ka: 0.752 (0.564–1.278) h^−1^; oral absorption lag time 2.29 (1.71–3.26) h; population parameter: 26% (9–59%). The differential proportional residual errors for whole blood sampling and DBS were 20.8% (17.3–23.9%) and 30.7% (23.4–37.9%), respectively. The IIV (CV%) for Ka was 60.5%. The inter-individual variability in CL/F and Vc/F were modeled as a variance–covariance matrix (BLOCK (2)) with a CV% of 32.1% for CL/F, 91.6% for Vc/F and 94% covariance between CL/F and Vc/F. Finally, the estimated IOV (CV%) for CL/F was 50.1%. A complete overview of the model parameters and the original model code is shown in Table 2 and Supplementary Data S1, respectively.

#### 3.3.2. Covariate Model

The a priori inclusion of allometric scaling improved the model by 9.7 points. Initial univariate covariate analyses revealed the significant (*p* < 0.05) effects of the hematocrit, CYP3A5 genotype and corticosteroid dose on CL. The effects of these covariates were as follows: (i) an increase in hematocrit was associated with a decreased CL, (ii) CYP3A5 expression was associated with a 62.5% increase in CL and (iii) clearance increased with an increasing corticosteroid dose. However, univariate analysis showed that hematocrit and prednisolone dose failed to explain the IIV for CL and that the effect of CYP3A5 expression on the IIV was minimal (4.4% decrease in IIV).

The stepwise covariate analysis retained hematocrit and CYP3A5 genotype for CL as covariates. However, since (i) hematocrit only reduced the clearance IOV (8.5% reduction) but not the IIV and (ii) CYP3A5 genotype reduced the clearance IIV by only 4.4%, we deemed these covariates to lack clinical significance. In addition, inclusion or exclusion of these covariates minimally affected the other parameter estimates (Table S2).

#### 3.3.3. Model Evaluation

**Figure 3:**
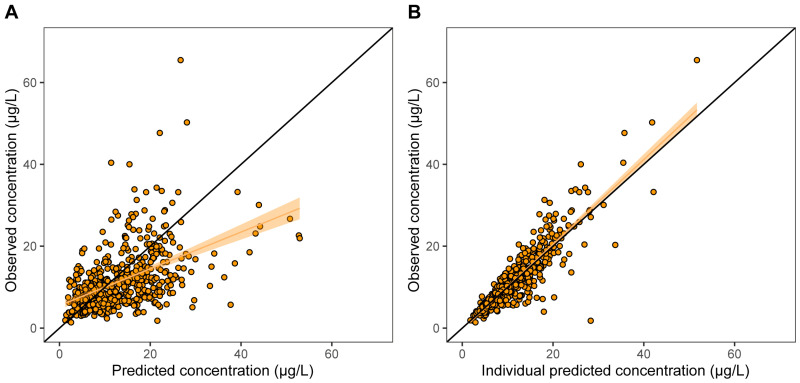
Basic goodness-of-fit plots. Observed tacrolimus concentrations vs. model-predicted concentrations (**A**) and observed tacrolimus concentrations vs. model individual-predicted concentrations (**B**).

The bootstrap median estimates were remarkably similar to the final model estimates, supporting the robustness of the model. Basic goodness-of-fit plots (Figure 3) showed that the model-predicted and individual-predicted concentrations were in acceptable agreement with the observed data. The NPDE VPC (Figure S1) indicated that the simulated data were adequately normally distributed. Additional individual model fits, AUC_0–24h_ vs. trough concentration plots and model AUC vs. trapezoidal AUC plots are shown in Figures S2–S4. In addition, the pcVPC with the Martial model [10] indicated that the Martial model was not able to fully describe our data. See also Figure S5.

#### 3.3.4. Time to Steady State Analysis

**Figure 4:**
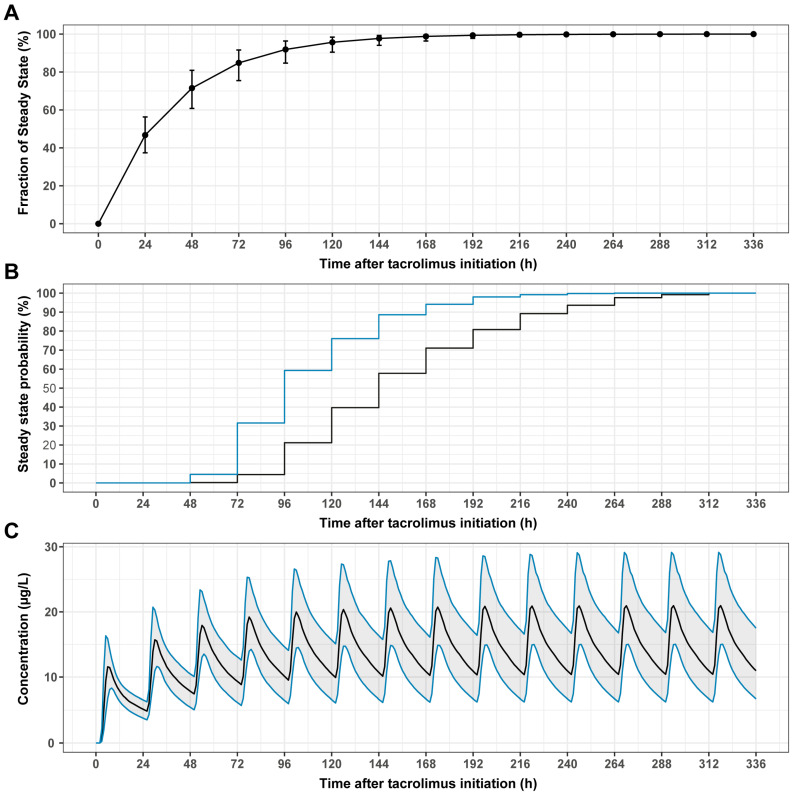
Time to steady-state tacrolimus concentrations. Median simulated fraction of the 90% steady state (IQR, error bars) vs. time after initiation of tacrolimus (**A**). Probability of reaching at least 90% (blue line) and 97% (black line) of the steady-state concentration vs. time after the start of tacrolimus dosing (**B**). Median simulated tacrolimus concentrations vs. time after tacrolimus initiation (IQR, shaded area) (**C**).

The simulation results for the time to steady state are shown in Figure 4. The median time to reach 90% and 97% of the assumed steady-state concentrations was 96 h (IQR: 72–120 h) and 144 h (IQR: 120–312 h), respectively. Five days after meltdose tacrolimus dose initiation, the probabilities of reaching 90% and 97% of the assumed steady-state concentration were 76% and 40%, respectively (Figure 4B). On day 7 after dose initiation, these probabilities were 94% and 71%, respectively.

#### 3.3.5. Limited Sampling Strategy

The best three-point LSS was T = 0, 2, 6 h, showing a mean relative prediction error of—1.9%, with a >10%, >15% and >20% prediction error in 2.3%, 0.6% and 0.4% of the cases, respectively. The best four-point LSSs were T = 0, 2, 6, 8 h and T = 0, 1, 6, 8 h, showing mean relative prediction errors of −1.8% and 1.8%, respectively. The T = 0, 2, 6, 8 h sampling scheme resulted in 0.4%, 0.2% and 0.0% of the cases with a >10%, >15% and >20% prediction error. Prediction errors >10%, >15% and >20% were observed in 1.2%, 0.2% and 0.0% of the cases with the T = 0, 1, 6, 8 h sampling scheme.

When using our model, the LSS reported by Woillard et al. (T = 0, 8, 12 h [11]) showed a mean relative prediction error of 10.8%, with a >20% relative prediction error in 16.6% of cases. In addition, the LSS reported by Martial et al. (T = 0, 4, 8 h [10]) showed a mean relative prediction error of 0.8%, with a >20% relative prediction error in 0.6% of cases. A complete overview of the limited sampling evaluation and concurrent metrics is shown in Table S3 and Figure S6.

## 4. Discussion

In the current study, we successfully developed a population PK model and LSS for meltdose tacrolimus in a cohort of elderly de novo kidney transplant recipients. In addition, we evaluated the clinical feasibility of outpatient DBS sampling. The outcomes of the current study can aid clinicians in individualizing meltdose tacrolimus dosing in elderly de novo kidney transplant recipients.

To our knowledge, only three other population PK models on meltdose tacrolimus are currently reported [10,11,21]. However, two of these reports were based on different populations (i.e., stable kidney transplant recipients [11] and liver transplant recipients [10]). In the first few months after kidney transplantation, the tacrolimus PK may change due to factors such as recovery (i.e., an increase) in the hematocrit values and tapering of the corticosteroid dosing. Therefore, a meltdose tacrolimus population PK model based on a stable kidney transplant population might not be suitable for this first phase after transplantation, where changes in pharmacokinetics are expected to occur.

The meltdose tacrolimus PK data in the current study were best described using a two-compartmental model, including three transit compartments and a mixture model for oral absorption. Whereas Martial et al. used a two-compartment model to describe the meltdose tacrolimus pharmacokinetics in a population of liver transplant recipients, Woillard et al. and Henin et al. described meltdose tacrolimus pharmacokinetics using a single compartment in populations of kidney transplant recipients [10,11,21]. Although we did evaluate a single-disposition-compartment model to fit our data, the addition of a second disposition compartment with a fixed peripheral volume of distribution parameters led to a significant (*p* < 0.01) improvement in the OFV.

The current model incorporated a mixture model for the absorption. Visual inspection of the data showed clear differences in the absorption lag times between patients. Previous studies showed that gastric emptying can be delayed in patients with diabetes [22]. We therefore hypothesized that the differences in the oral absorption lag time between patients might be related to diabetic gastroparesis. In the early stages of model building, diabetes was included in a simple two-compartment (pilot) model with the absorption lag time. However, no significant effect of diabetes was shown on the absorption lag parameter.

Older kidney transplant recipients have shown different outcomes compared to younger populations when it comes to the risk of acute graft rejection and the occurrence of infections or malignancies [14]. We postulated that these differences in clinical outcomes might partially be explained by changes in the tacrolimus pharmacokinetics, associated with the process of aging. In a cohort of 55 liver transplant patients (median [range] age: 57 years [21–70 years]), Martial et al. reported an apparent elimination clearance of 3.27 L/h at 70 kg with the assumption of an oral bioavailability of 23% [10]. Recalculating this clearance to an oral bioavailability of 100%, this apparent clearance would be 14.2 L/h at 70 kg. In addition, in a population of 33 de novo kidney transplant recipients (median [range] age: 57 years [32–81 years]), Henin et al. reported an apparent elimination clearance of 20 L/h at 70 kg [21]. Since the apparent meltdose tacrolimus elimination clearance in the current model was 19.6 L/h at 70 kg, no substantial differences in the model parameters were observed compared to the population PK models based on younger populations.

We did not observe clinically relevant covariate effects for the meltdose tacrolimus pharmacokinetics. Although genetic status and hematocrit led to improvements in the model’s OFV, the effect on the interindividual variability of the elimination clearance was neglectable. Interestingly, the outcomes of the covariate analysis in the study from Martial et al. were comparable to our findings, despite using a larger cohort compared to the current study (*n* = 55) [10]. On the contrary, Henin et al. were able to identify a significant effect of CYP3A5 status on elimination clearance in a population of 33 de novo kidney transplant recipients [21]. Based on a previously published study, we assumed a population of approximately 35 patients should be sufficient to reliability identify a covariate effect of 50% on elimination clearance [15]. Although we cannot exclude the possibility that our dataset lacked the power to identify smaller covariate effects (i.e., <50% on elimination clearance), we did not consider this to be a limitation of the current study since the clinical relevance of such smaller covariate effects would be neglectable.

Another important aim of this study was to assess the practical feasibility of using DBS sampling in outpatient settings. Our evaluation showed 42.5% of the AUCs were obtained using DBS sampling. However, only 54% of these DBS AUCs were sampled exactly in conformance with protocol. Although no data on the causes of outpatient DBS sampling errors were available in the current study, a previous study emphasized the role of proper patient training and guidance, potentially affecting both the correct timing and execution of the sampling [16].

Evaluation of the time to steady state suggested that at least five to seven days are needed to achieve a 76% and 94% probability of reaching 90% of the steady state, respectively. These findings are further supported by the data from the FDA-approved drug label [12]. Based on these findings, we suggest the first sampling of conventional trough concentrations to be performed not earlier than on the 5th day and preferably on day 7 or later after the start of meltdose tacrolimus. However, using MIPD methods in combination with early sampled AUC data (e.g., sampled on day two after start) might enable clinicians to estimate the tacrolimus exposure at the steady state, and thus perform early dose adjustments if necessary.

Several limited sampling strategies were evaluated. Using the best three- and four-point sampling schemes (i.e., 0, 2, 6 h; 0, 2, 6, 8 h and 0, 1, 2, 8 h), the AUCs could be estimated with acceptable bias. The bias percentage and percentage of the population with >20% deviation from the reference AUC were like that of the limited sampling strategies reported by Martial et al. [10]. However, Martial et al. reported sampling schemes at t = 0, 4, 8 h and t = 0, 3, 6, 8 h to be the optimal sampling times. This difference might be explained by subtle differences in the absorption models, causing differences in the early-phase pharmacokinetics. Although the three-point sampling scheme proposed by Martial et al. resulted in a remarkably similar predictive performance to our best three-point sampling scheme, the 6 h sampling interval described in the current study might be more convenient for an outpatient setting. On the contrary, the LSS proposed by Woillard et al. [11] resulted in a lower predictive performance compared to our 0, 2, 6 h sampling scheme, when using the current population pharmacokinetic model. This difference may be caused by the structural differences between both models and study populations.

We know our study has one important limitation that must be addressed. Since our data were confined to de novo kidney transplant recipients who were 65 years or older, this limitation of the age group might have contributed to the fact that we were unable to identify a potential age effect on the tacrolimus pharmacokinetics in our model. Although a comparison of our model parameters with those of previously published meltdose tacrolimus models in younger populations [10,11,21] did not suggest differential pharmacokinetics of meltdose tacrolimus in elderly patients, studies including a wider age range will be necessary to further elucidate a potential age effect.

## 5. Conclusions

In the current study, a population pharmacokinetics model for meltdose tacrolimus was developed in elderly kidney transplant patients. The model parameters were comparable to those of previously reported models in younger populations. Furthermore, simulations suggested a prolonged time to reach steady-state pharmacokinetics with meltdose tacrolimus. The observed high inter-individual variability in the meltdose tacrolimus pharmacokinetics stresses the need for MIPD for its effective and safe use. Finally, the proposed limited sampling strategies predicted the true AUC with sufficient accuracy to be used in clinical practice. The developed population PK model and LSS may aid clinicians in the dose individualization of meltdose tacrolimus in de novo elderly kidney transplant recipients. Future studies might be directed toward elucidating the effect of patient characteristics such as age, CYP3A5 genetic status or hematocrit on the meltdose tacrolimus pharmacokinetics.

## Figures and Tables

**Table 1:** Patient characteristics.

**Demographics**	
Age (median [IQR])	71.5 [68.8–73.2]
Female (n, [%])	12 [35.3%]
Weight (kg, median [IQR])	77.0 [68.1–84.5]
BMI (kg/m^2^, median [IQR])	25.9 [23.3–28.0]
Days post kTx for first AUC (median [IQR])	7.0 [5.0–9.0]
Prednisolone dose (mg, median [range])	10 [5–50]
Calcium blocker use (n [%])	20 [58.8%]
Diabetes (n [%])	14 [41.2%]
**Primary diagnosis**	
Renal	
ADPKD	6
Glomerulonephritis	8
Urologic, reflux	3
Diabetic nephropathy	5
Secondary FSGS	5
Benign neoplasma	1
Malignancy	1
Post renal	1
Kidney failure of unknown origin	4
**Pharmacokinetic data**	
Samples total (n)	546
Total number of AUCs (n)	87
AUCs per subject (n, median [range])	2 [1–5]
DBS AUCs (n [%])	37 [42.5%]
Patients with DBS sampling (n [%])	20 [58.8%]
Tacrolimus dose (mg, median [IQR])	6.0 [3.25–7.0]
**Clinical chemistry**	
Hematocrit (L/L, median [IQR])	0.334 [0.297–0.362]
Creatinine (µmol/L, median [IQR])	132 [99–177]
Albumin (g/L, median [IQR])	40 [36–44]
ASAT (U/L, median [IQR])	25.5 [19.3–35.0]
Bilirubin (µmol/L, median [IQR])	5 [4–7]
**CYP3A5 status**	
CYP3A5*3/*3 (n, [%])	26 [76.5%]
CYP3A5*1/*3 (n, [%])	6 [17.6%]
CYP3A5*1/*1 (n, [%])	2 [5.9%]

BMI, Body Mass Index; AUC, Area Under the Curve; ADPKD, Autosomal Dominant Polycystic Kidney Disease; FSGS, Focal Segmental Glomerulosclerosis; DBS, Dried Blood Spot; ASAT, Aspartate Aminotransferase; CYP3A5, Cytochrome P450 3A5.

**Table 2:** Population pharmacokinetic model parameters.

	Final Model			Bootstrap	
Parameter	Estimate	RSE (%)	Shrinkage (%)	Median Estimate	95% Bootstrap Confidence Interval
K_tr_ (h^−1^)	0.752	21	-	0.807	0.564–1.278
Lag (h)	2.29	2	-	2.31	1.71–3.26
CL/F (L/h at 70 kg)	19.6	8	-	19.6	16.4–22.8
Q/F (L/h at 70 kg)	74.9	9	-	73.1	55.8–86.5
Vc/F (L per 70 kg)	123	38	-	146	65–273
Vp/F (L per 70 kg) (Fixed)	500	-	-	500	500
Pop parameter (%)	26	50	-	28	9–59
F (Fixed)	1	-	-	1	1
Interindividual variability					
Ka (CV%)	60.5	19	0	61.2	30.0–96.4
CL (CV%)	32.1	26	20	30.4	10.0–49.2
Vc (CV%)	91.6	23	16	85.4	14–137
Interoccasion variability					
CL (CV%)	50.1	12	33; 13; 77; 100; 100	49.8	37.4–137.3
Proportional Error					
Whole blood (%)	20.8	8	-	20.3	17.3–23.9
DBS (%)	30.7	12	-	30.6	23.4–37.9

K_tr_, oral transit constant; Lag, oral absorption lag time; CL/F, apparent elimination clearance; Q/F, apparent intercompartmental clearance; Vc/F, apparent central volume of distribution; Vp/F, apparent peripheral volume of distribution; Pop parameter, proportion of the population belonging to the group without oral lag time assigned by the mixture model; F, oral bioavailability; RSE (%), relative standard error; CV%, coefficient of variation; DBS, dried blood spot sampling.

## Data Availability

Original data are available on request.

## Supplementary Materials

The following supporting information can be downloaded at https://www.mdpi.com/article/10.3390/pharmaceutics16010017/s1, Figure S1: Normalized Prediction Distribution Error plots (NDPE); Figure S2: Individual model fits. Blue and red dots show the observed whole blood and DBS concentrations respectively; Figure S3: Calculated tacrolimus AUC_0-24h_ vs. trough concentrations; Figure S4: Passing-Bablok analysis of model AUC_0-24h_ vs. trapezoidal AUC_0-24h_; Figure S5: Prediction corrected visual predictive check (pcVPC) of our data with the Martial model (9); Figure S6: Limited sampling reference AUC_0-24_ vs. the AUC_0-24_ estimated by the reduced limited sampling datasets; Table S1: Overview of the expected versus observed DBS sampling times (1); Table S2: Effects of covariates on model parameters; Table S3: Overview of the tested limited sampling strategies; Supplementary data S1: NONMEM code.

## References

[1] Cossart A.R., Cottrell W.N., Campbell S.B., Isbel N.M., Staatz C.E. (2019). Characterizing the pharmacokinetics and pharmacodynamics of immunosuppressant medicines and patient outcomes in elderly renal transplant patients. Transl. Androl. Urol..

[2] Lentine K.L., Smith J.M., Miller J.M., Bradbrook K., Larkin L., Weiss S., Handarova D.K., Temple K., Israni A.K., Snyder J.J. (2023). OPTN/SRTR 2021 Annual Data Report: Kidney. Am. J. Transplant..

[3] Brunet M., van Gelder T., Asberg A., Haufroid V., Hesselink D.A., Langman L., Lemaitre F., Marquet P., Seger C., Shipkova M. (2019). Therapeutic Drug Monitoring of Tacrolimus-Personalized Therapy: Second Consensus Report. Ther. Drug Monit..

[4] Darwich A.S., Polasek T.M., Aronson J.K., Ogungbenro K., Wright D.F.B., Achour B., Reny J.L., Daali Y., Eiermann B., Cook J. (2021). Model-Informed Precision Dosing: Background, Requirements, Validation, Implementation, and Forward Trajectory of Individualizing Drug Therapy. Annu. Rev. Pharmacol. Toxicol..

[5] Marquet P., Bedu A., Monchaud C., Saint-Marcoux F., Rerolle J.P., Etienne I., Kamar N., Moulin B., Cassuto E., Essig M. (2018). Pharmacokinetic Therapeutic Drug Monitoring of Advagraf in More Than 500 Adult Renal Transplant Patients, Using an Expert System Online. Ther. Drug Monit..

[6] Saint-Marcoux F., Woillard J.B., Jurado C., Marquet P. (2013). Lessons from routine dose adjustment of tacrolimus in renal transplant patients based on global exposure. Ther. Drug Monit..

[7] Meziyerh S., van Gelder T., Kers J., van der Helm D., van der Boog P.J.M., de Fijter J.W., Moes D., de Vries A.P.J. (2023). Tacrolimus and Mycophenolic Acid Exposure Are Associated with Biopsy-Proven Acute Rejection: A Study to Provide Evidence for Longer-Term Target Ranges. Clin. Pharmacol. Ther..

[8] Zwart T.C., Guchelaar H.J., van der Boog P.J.M., Swen J.J., van Gelder T., de Fijter J.W., Moes D. (2021). Model-informed precision dosing to optimise immunosuppressive therapy in renal transplantation. Drug Discov. Today.

[9] Garnock-Jones K.P. (2015). Tacrolimus prolonged release (Envarsus(R)): A review of its use in kidney and liver transplant recipients. Drugs.

[10] Martial L.C., Biewenga M., Ruijter B.N., Keizer R., Swen J.J., van Hoek B., Moes D. (2021). Population pharmacokinetics and genetics of oral meltdose tacrolimus (Envarsus) in stable adult liver transplant recipients. Br. J. Clin. Pharmacol..

[11] Woillard J.B., Debord J., Monchaud C., Saint-Marcoux F., Marquet P. (2017). Population Pharmacokinetics and Bayesian Estimators for Refined Dose Adjustment of a New Tacrolimus Formulation in Kidney and Liver Transplant Patients. Clin. Pharmacokinet..

[12] FDA Envarsus Prescribing Information 2018. https://www.accessdata.fda.gov/drugsatfda_docs/label/2018/206406s007lbl.pdf.

[13] Zwart T.C., Gokoel S.R.M., van der Boog P.J.M., de Fijter J.W., Kweekel D.M., Swen J.J., Guchelaar H.J., Moes D. (2018). Therapeutic drug monitoring of tacrolimus and mycophenolic acid in outpatient renal transplant recipients using a volumetric dried blood spot sampling device. Br. J. Clin. Pharmacol..

[14] de Boer S.E., Sanders J.S.F., Bemelman F.J., Betjes M.G.H., Burgerhof J.G.M., Hilbrands L., Kuypers D., van Munster B.C., Nurmohamed S.A., de Vries A.P.J. (2021). Rationale and design of the OPTIMIZE trial: OPen label multicenter randomized trial comparing standard IMmunosuppression with tacrolimus and mycophenolate mofetil with a low exposure tacrolimus regimen In combination with everolimus in de novo renal transplantation in Elderly patients. BMC Nephrol..

[15] Press R.R., Ploeger B.A., den Hartigh J., van der Straaten T., van Pelt J., Danhof M., de Fijter J.W., Guchelaar H.J. (2009). Explaining variability in tacrolimus pharmacokinetics to optimize early exposure in adult kidney transplant recipients. Ther. Drug Monit..

[16] Zwart T.C., Metscher E., van der Boog P.J.M., Swen J.J., de Fijter J.W., Guchelaar H.J., de Vries A.P.J., Moes D. (2022). Volumetric microsampling for simultaneous remote immunosuppressant and kidney function monitoring in outpatient kidney transplant recipients. Br. J. Clin. Pharmacol..

[17] Bergstrand M., Hooker A.C., Wallin J.E., Karlsson M.O. (2011). Prediction-corrected visual predictive checks for diagnosing nonlinear mixed-effects models. AAPS J..

[18] Khandelwal A., Harling K., Jonsson E.N., Hooker A.C., Karlsson M.O. (2011). A fast method for testing covariates in population PK/PD Models. AAPS J..

[19] Savic R.M., Jonker D.M., Kerbusch T., Karlsson M.O. (2007). Implementation of a transit compartment model for describing drug absorption in pharmacokinetic studies. J. Pharmacokinet. Pharmacodyn..

[20] Benkali K., Rostaing L., Premaud A., Woillard J.B., Saint-Marcoux F., Urien S., Kamar N., Marquet P., Rousseau A. (2010). Population pharmacokinetics and Bayesian estimation of tacrolimus exposure in renal transplant recipients on a new once-daily formulation. Clin. Pharmacokinet..

[21] Henin E., Govoni M., Cella M., Laveille C., Piotti G. (2021). Therapeutic Drug Monitoring Strategies for Envarsus in De Novo Kidney Transplant Patients Using Population Modelling and Simulations. Adv. Ther..

[22] Bharucha A.E., Kudva Y.C., Prichard D.O. (2019). Diabetic Gastroparesis. Endocr. Rev..

