# Supplementary material for: Meltdose Tacrolimus Population Pharmacokinetics and Limited Sampling Strategy Evaluation in Elderly Kidney Transplant Recipients
Source: Pharmaceutics. 2023 Dec 21;16(1):17. doi: 10.3390/pharmaceutics16010017 (PMC10819724; doi:10.3390/pharmaceutics16010017)
Supplement: Supplementary file 1 [file pharmaceutics-16-00017-s001.zip › pharmaceutics-2748522-supplementary.pdf]

Supplementary data

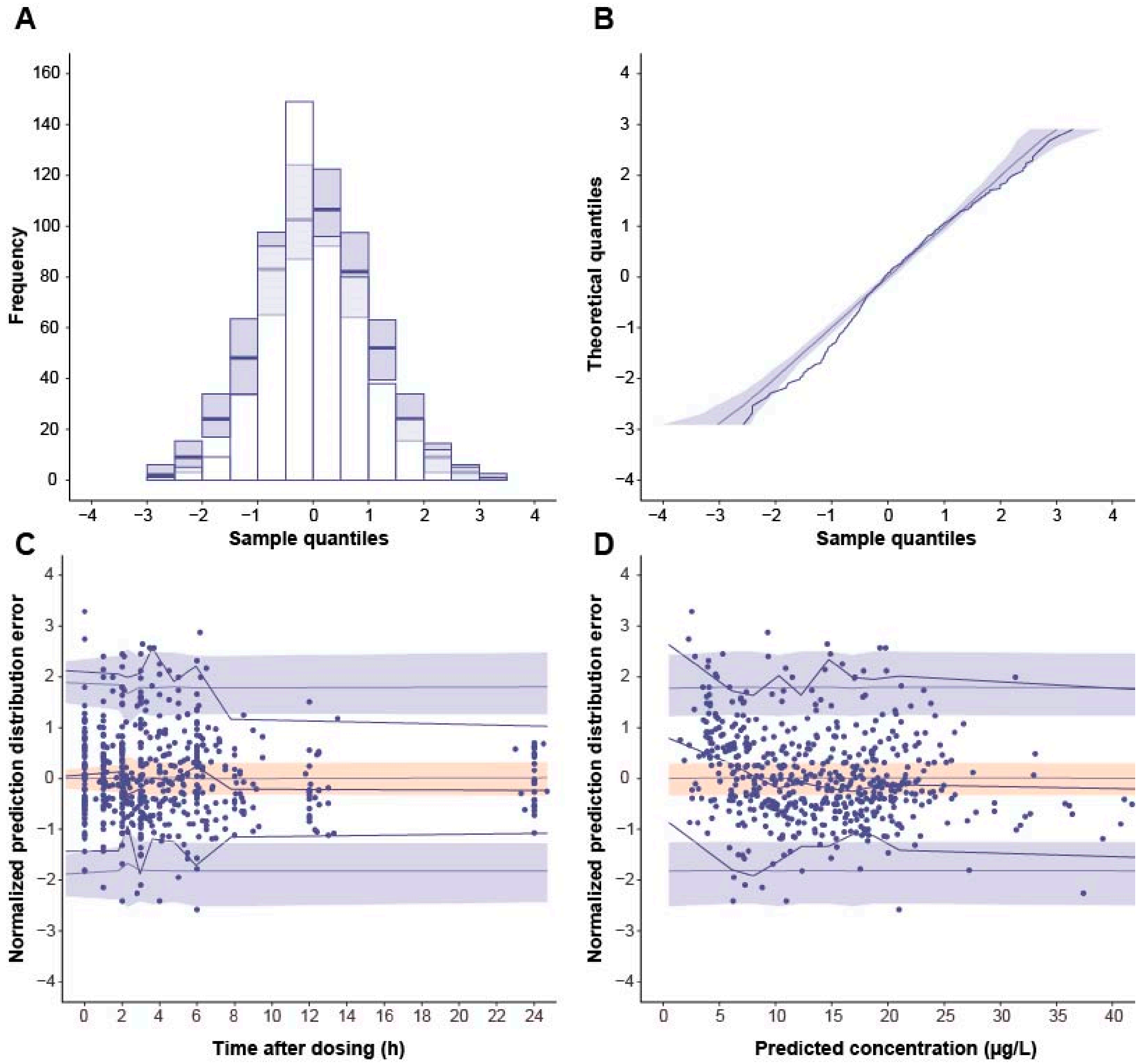

Figure S1: Normalized Prediction Distribution Error plots (NDPE). Distribution of NPDE quantiles (A); Theoretical NPDE quantiles vs. Sample quantiles (B); NPDE VPC: NPDE vs. Time (C); NPDE VPC: NPDE vs. model predicted concentration (D)

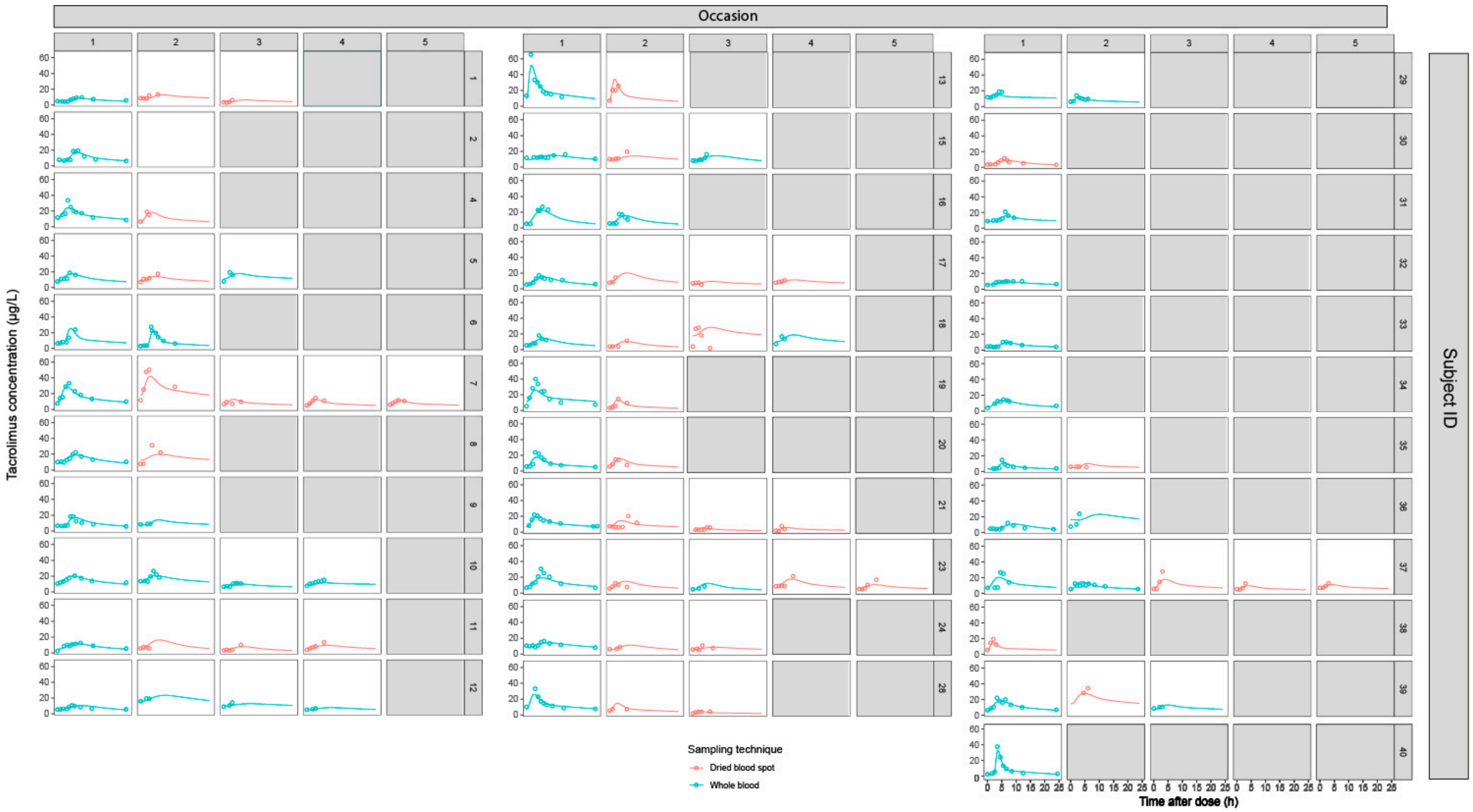
Occasion
1
2
3
4
5
Subject ID
Tacrolimus concentration (µg/L)
Time after dose (h)
Sampling technique
Dried blood spot
Whole blood

Figure S2: Individual model fits. Blue and red dots show the observed whole blood and DBS concentrations respectively. The blue and red lines show the individual predicted concentration profiles for the whole blood concentrations and DBS concentrations respectively. Concentration time profiles are shown for each subject for each separate occasion.

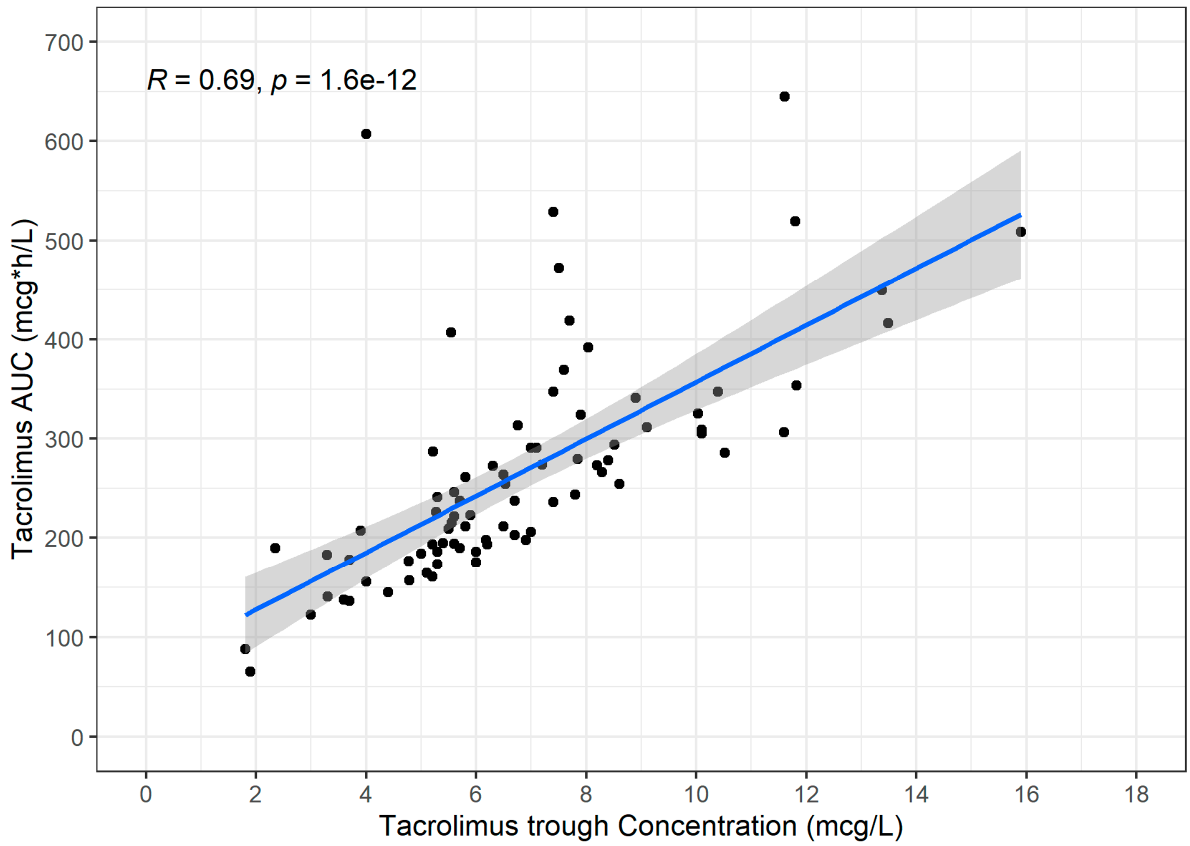

Figure S3: Calculated tacrolimus $AUC_{0\text{-}24h}$ vs. trough concentrations. Model $AUC_{0\text{-}24h}$ was calculated as $AUC_{0\text{-}24h}$ = (F * D * 1000) / CL, where F is the oral bioavailability (fixed to 100%), D the drug dose in mg and CL the apparent elimination clearance in L/h.

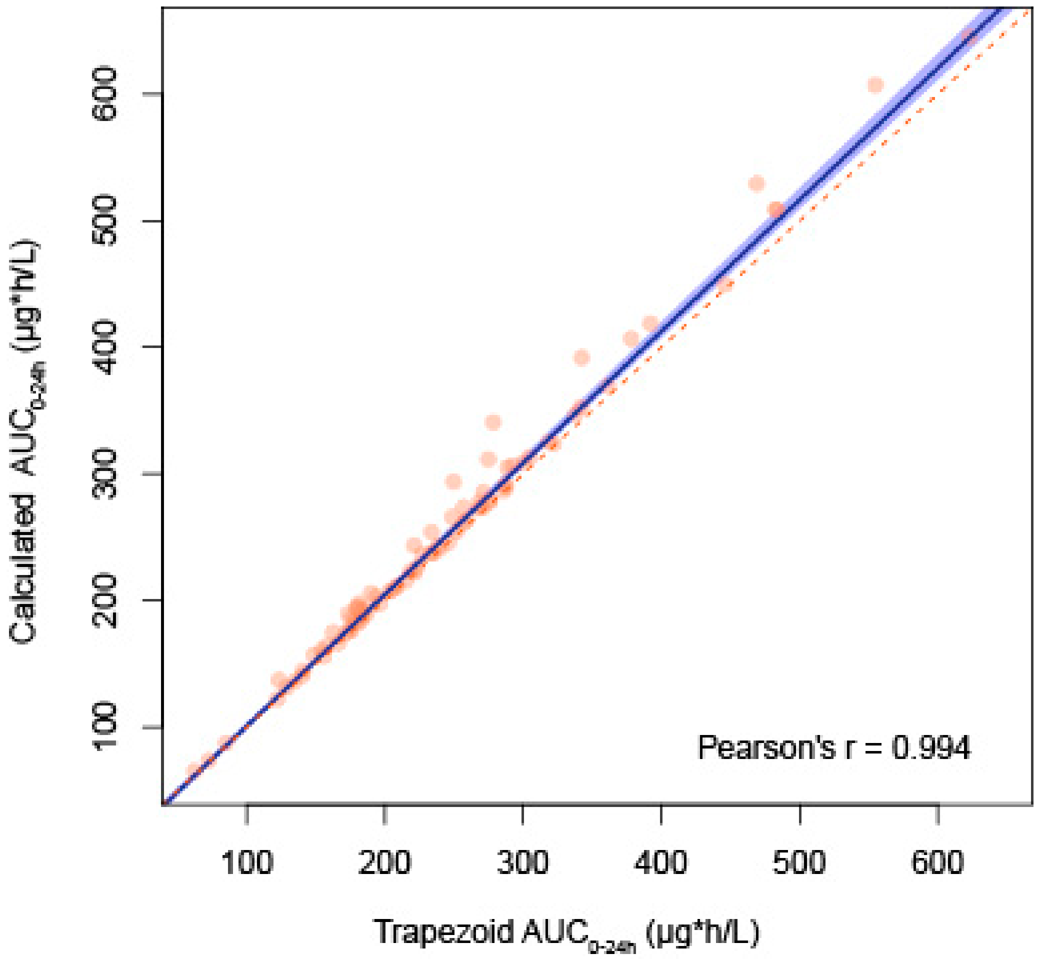

Figure S4: Passing-Bablok analysis of model $AUC_{0\text{-}24h}$ vs. trapezoidal $AUC_{0\text{-}24h}$. Model $AUC_{0\text{-}24h}$ was calculated as $AUC_{0\text{-}24h}$ = (F * D * 1000) / CL, where F is the oral bioavailability (fixed to 100%), D the drug dose in mg and CL the apparent elimination clearance in L/h.

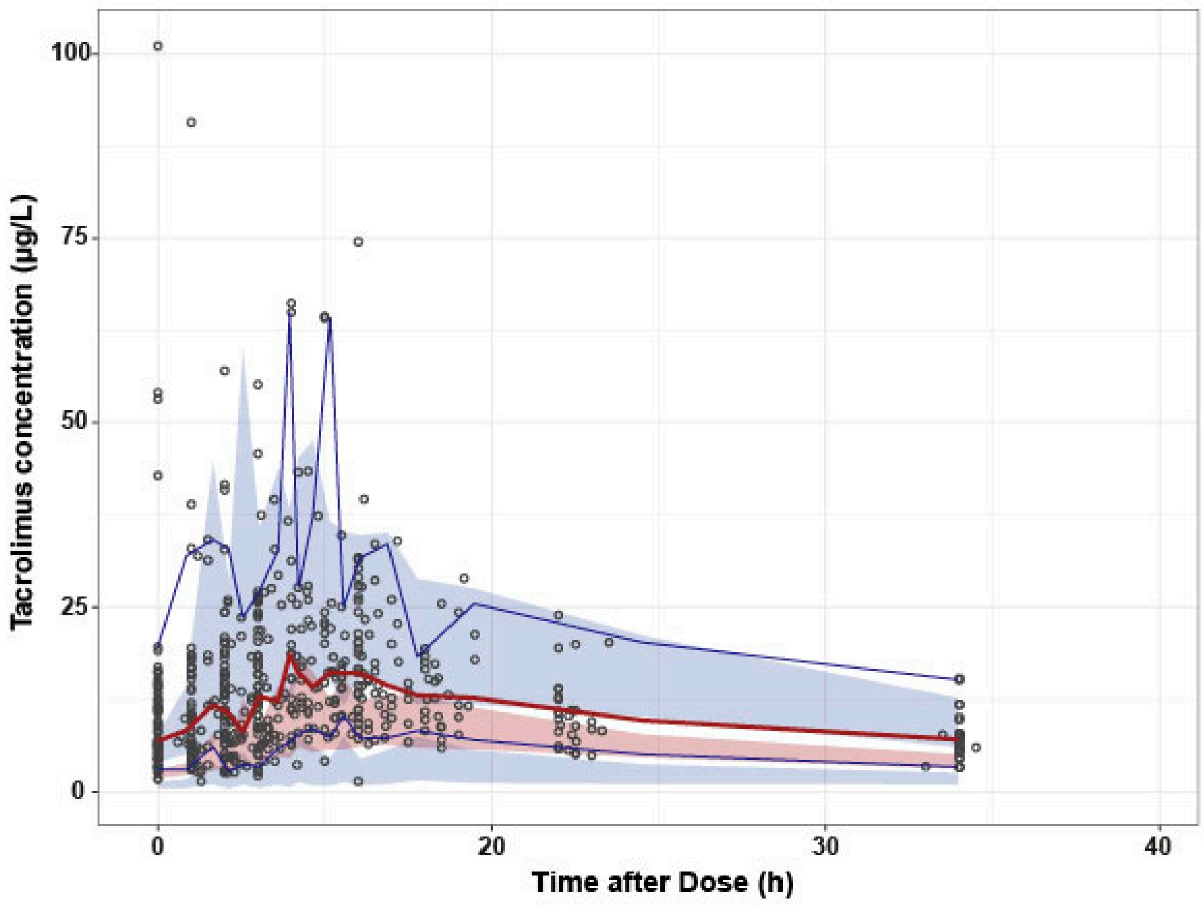

Figure S5: Prediction corrected visual predictive check (pcVPC) of our data with the Martial model (9). The observed prediction corrected datapoints are shown by the dots. The lower and upper blue lines show the 5th and 95th percentiles of the observed data. The 50th percentile of the observed data is shown by the red line. The 95% prediction intervals of the simulated 5th, 95th and 50th percentiles are shown by the blue and red shaded areas respectively.

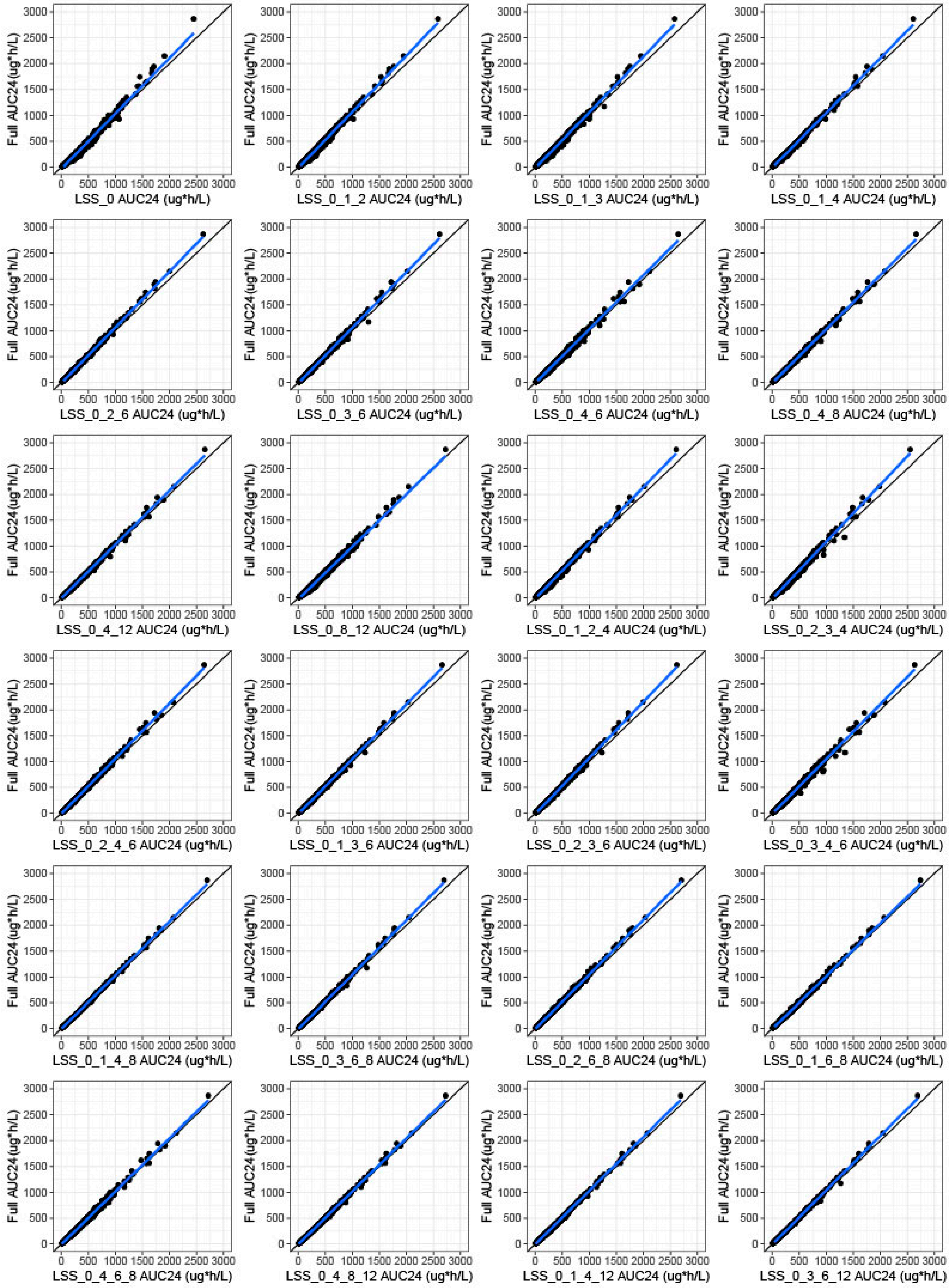

Figure S6: Limited sampling reference AUC_{0-24} vs. the AUC_{0-24} estimated by the reduced limited sampling datasets. The 95% confidence intervals are shown by the gray shaded areas. Note however, that the confidence interval is almost invisible because of the extremely narrow 95% confidence intervals. The included limited sampling timepoints in hours after dose are shown in the x-axis title.

Table S1: Overview of the expected versus observed DBS sampling times(1)

Sampling Time (h)	Number of samples	Frequency (%)
0†	37	100.0
1†	33	89.2
2†	38	102.7‡
3†	34	91.9
4	7	18.9
5	4	10.8
6†	22	59.5
7	2	5.4
10	1	2.7

†Sampling scheme as according to protocol. ‡One patient drew 2 samples at approximately the same sampling time. The frequency is defined as the proportion of DBS AUCs in which the designated timepoint was sampled. E.g.: 100% of the DBS AUCs included a T=0h sample. However, only 59.5% of the DBS AUCs included a T=6 h sample.

Table S2: Effects of covariates on model parameters

	Final model		Covariate model	
**Parameter**	**Estimate**	**Shrinkage (%)**	**Estimate**	**Shrinkage (%)**
K_{tr} (h^{-1})	0.752	-	0.758	-
Lag (h)	2.29	-	2.28	-
CL/F (L/h at 70 kg)	19.6	-	17.2	-
Q/F (L/h at 70 kg)	74.9	-	73.5	-
Vc/F (L per 70 kg)	123	-	128	-
Vp/F (L per 70 kg) (Fixed)	500	-	500	-
Pop parameter (%)	26	-	25.7	-
F (Fixed)	1	-	1	-
Covariate effects				
CL - hematocrit	-	-	-4.94	-
CL - CYP3A5 expressor	-	-	0.625	-
Interindividual variability				
Ka (CV%)	60.5	0	57.8	0
CL (CV%)	32.1	20	25.5	22
Vc (CV%)	91.6	16	96.7	18
Interoccasion variability				
CL [Block] (CV%)	50.1	33; 13; 77; 100; 100	41.7	25; 27; 79; 100
Proportional Error				
Whole blood (%)	20.8	-	20.9	-
DBS (%)	30.7	-	30.6	-

K_{tr}, oral transit constant; Lag, oral absorption lag time; CL/F, apparent elimination clearance; Q/F, apparent intercompartmental clearance; Vc/F, apparent central volume of distribution; Vp/F, apparent peripheral volume of distribution; Pop parameter, proportion of the population belonging to the group without oral lag time assigned by the mixture model; F, oral bioavailability; CV%, coefficient of variation; DBS, Dried Blood Spot sampling.

Table S3: Overview of the tested limited sampling strategies.

Sampling times (h)	R^2	>10%	>15%	>20%	Mean PPE (%)	Median PPE (%)	Mean APE	Median APE
**Trough concentration**								
0	0.986	36.0	23.9	15.1	9.140	5.883	12.132	11.630
**Three sample limited sampling schemes**								
0-2-6*	0.998	2.3	0.6	0.4	-1.850	-2.516	-11.873	-5.140
0-4-8†	0.996	4.4	1.6	0.6	0.795	0.157	-3.088	0.309
0-4-12	0.997	5.2	1.8	0.7	0.203	-1.337	-4.155	-2.020
0-3-6	0.996	4.6	1.5	0.9	-0.530	-0.735	-9.235	-1.365
0-4-6	0.992	11.3	4.3	2.0	1.399	0.294	-2.400	0.386
0-1-4	0.996	6.0	3.6	2.2	-0.490	-2.871	-7.494	-5.115
0-1-2	0.992	13.6	8.6	5.3	0.510	-2.543	-9.516	-4.865
0-1-3	0.991	15.6	8.5	5.3	1.338	0.734	-5.938	0.992
0-8-12‡	0.992	42.3	25.7	16.6	10.832	8.292	20.826	19.745
**Four sample limited sampling schemes**								
0-2-6-8*	0.998	0.4	0.2	0.0	-1.806	-1.701	-9.674	-3.601
0-1-6-8*	0.998	1.2	0.2	0.0	1.820	1.789	1.942	3.617
0-1-4-8	0.999	1.1	0.4	0.1	-0.332	-0.942	-4.934	-1.850
0-4-8-12	0.998	1.7	0.2	0.1	0.664	0.032	-1.360	0.065
0-3-6-12	0.998	0.7	0.4	0.1	-0.744	-1.186	-6.981	-1.880
0-3-6-8	0.998	0.8	0.4	0.2	-1.091	-1.189	-8.158	-2.475
0-1-4-12	0.998	3.7	1.4	0.3	-0.495	-2.167	-5.261	-4.065
0-2-4-6	0.997	2.0	1.1	0.4	-1.782	-2.366	-11.709	-4.248
0-1-3-6	0.998	2.8	1.3	0.6	-0.412	-0.844	-7.283	-1.451
0-2-3-6	0.996	3.0	1.4	0.8	-1.564	-1.031	-12.408	-2.010
0-4-6-8	0.995	9.5	2.2	0.9	1.798	1.387	0.605	2.254
0-3-4-6	0.992	5.4	2.3	1.2	-0.399	0.230	-8.416	0.335

0-1-2-4	0.996	7.1	4.4	2.6	-1.257	-4.238	-11.526	-8.180
0-2-3-4	0.991	8.3	4.9	2.8	-1.233	-0.243	-14.927	-0.260

R^2, correlation coefficient; >10 %, >15% >20%, proportions of the simulated AUCs with > 10%, 15% or 20% bias respectively; PPE, percentage prediction error; APE, absolute prediction error. *Best sampling scheme based on the current model. †Sampling scheme as reported by Martial et al.; ‡Sampling scheme as reported by Woillard et al.

Supplementary data S1: NONMEM code

```
$SUBROUTINES ADVAN6 TOL=6
;------------------------------------------------------------------------------------------------------------------------------------
$ABBREVIATED REPLACE ETA(OCC_CL)=ETA(4,5,6,7,8)
;------------------------------------------------------------------------------------------------------------------------------------
$MIX
P(1) = THETA(11)
P(2) = 1.0 - THETA(11)
NSPOP = 2
;------------------------------------------------------------------------------------------------------------------------------------
$MODEL
COMP =(TRANS1, DEFDOSE) ; Absorption compartment 1
COMP =(TRANS2)          ; Absorption compartment 2
COMP =(TRANS3)          ; Absorption compartment 3

COMP=(CENTRAL, DEFOBS)  ; Central volume of distribution
COMP=(PERIPH)           ; Peripheral volume of distribution
;------------------------------------------------------------------------------------------------------------------------------------
$PK
IF (AMT.GT.0) THEN
TDOS=TIME
TAD=0.0
ENDIF
IF (AMT.EQ.0) TAD=TIME-TDOS
IF (TIME.EQ.120) TAD=0

TVKA  = THETA(6)
TVCL  = THETA(7)
TVV2  = THETA(8)
```

```
TVQ     = THETA(9)
TVV3   = THETA(10)

IF(MIXNUM.EQ.2) THEN
ALAG1 = THETA(4)
ELSE
ALAG1 = THETA(5)
ENDIF
BESTSUB=MIXEST

ALLO_V = WT/70
ALLO_CL = ALLO_V ** 0.75

KA = TVKA  * EXP(ETA(1))
CL  = TVCL  *  ALLO_CL * EXP(ETA(2) + ETA(OCC_CL))
V2  = TVV2  * ALLO_V *  EXP(ETA(3))
Q   = TVQ    *  ALLO_CL
V3  = TVV3 *  ALLO_V
S4 = V2/1000

AUCCL = (AMT*1000/CL)

K12 = KA
KTR =  KA
K23 = KTR
K34 = KTR

K40 = CL/V2
K45 = Q/V2
K54 = Q/V3
```

```
$DES
DADT(1) =  -KA*A(1)
DADT(2) =  KA*A(1) - KTR*A(2)
DADT(3) =  KTR*A(2) - KTR*A(3)

DADT(4) =  KTR*A(3) - K45*A(4) + K54*A(5) - K40*A(4)
DADT(5) =  K45*A(4) - K54*A(5)
;-----------------------------------------------------------------------------------------------------------------------------
$THETA
0.208 ; Whole blood proportional residual error
0.307 ; DBS proportional residual error
0.0001 FIX ; additive error
0 FIX  ; Absorption lag time for group 1
2.29  ; Absorption lag time for group 2
19.6  ; Elimination clearance
123   ; Central volume of distribution
74.9  ; Intercompartmental clearance
500 FIX ;  Peripheral volume of distribution
0.26 ; Population parameter
;-----------------------------------------------------------------------------------------------------------------------------
$OMEGA
 0.312 ; IIV Absorption rate constant

$OMEGA BLOCK(2)
0.0979  ; IIV elimination clearance
0.23 0.609  ; IIV Central volume of distribution

$OMEGA BLOCK(1) 0.224  ; IOV elimination clearance
$OMEGA BLOCK(1) SAME
$OMEGA BLOCK(1) SAME
$OMEGA BLOCK(1) SAME
```

```
$OMEGA BLOCK(1) SAME
;-----------------------------------------------------------------------------------------------------------------------------------
$SIGMA
 1 FIX  ; residual variability
;-----------------------------------------------------------------------------------------------------------------------------------
$ERROR
IF(DBS0.EQ.1) THEN
PROPERR = THETA(1)
ELSE
PROPERR = THETA(2)
ENDIF

IPRED = F
IRES = DV-IPRED
W = SQRT(PROPERR**2*IPRED**2 + THETA(3)**2)
IF (W.EQ.0) W = 1
IWRES = IRES/W
Y= IPRED+W*ERR(1)
;-----------------------------------------------------------------------------------------------------------------------------------
$EST METHOD=1 INTERACTION MAXEVAL=9999 NSIG=3 SIGL=9 PRINT=1 NOABORT
NOTHETABOUNDTEST POSTHOC
;-----------------------------------------------------------------------------------------------------------------------------------
$COV PRINT=E
;-----------------------------------------------------------------------------------------------------------------------------------
```
